# Machine Learning–Based Predictive Modeling of Anxiety and Depressive Symptoms During 8 Months of the COVID-19 Global Pandemic: Repeated Cross-sectional Survey Study

**DOI:** 10.2196/32876

**Published:** 2021-11-17

**Authors:** Katrina Hueniken, Nibene Habib Somé, Mohamed Abdelhack, Graham Taylor, Tara Elton Marshall, Christine M Wickens, Hayley A Hamilton, Samantha Wells, Daniel Felsky

**Affiliations:** 1 Krembil Centre for Neuroinformatics Centre for Addiction and Mental Health Toronto, ON Canada; 2 Dalla Lana School of Public Health University of Toronto Toronto, ON Canada; 3 Institute for Mental Health Policy Research Centre for Addiction and Mental Health Toronto, ON Canada; 4 Campbell Family Mental Health Research Institute Centre for Addiction and Mental Health Toronto, ON Canada; 5 Institute for Clinical Evaluative Sciences Toronto, ON Canada; 6 Department of Epidemiology and Biostatistics Schulich School of Medicine and Dentistry Western University London, ON Canada; 7 School of Engineering University of Guelph Guelph, ON Canada; 8 Vector Institute for Artificial Intelligence Toronto, ON Canada; 9 School of Epidemiology and Public Health, Faculty of Medicine University of Ottawa Ottawa, ON Canada; 10 Department of Health Sciences Lakehead University Thunder Bay, ON Canada; 11 Institute of Health Policy, Management and Evaluation University of Toronto Toronto, ON Canada; 12 Department of Pharmacology and Toxicology University of Toronto Toronto, ON Canada; 13 Department of Psychiatry University of Toronto Toronto, ON Canada; 14 School of Psychology Deakin University Burwood Australia; 15 Department of Biostatistics Dalla Lana School of Public Health University of Toronto Toronto, ON Canada; 16 Institute of Medical Sciences University of Toronto Toronto, ON Canada

**Keywords:** mental health, machine learning, COVID-19, emotional distress, emotion, distress, prediction, model, anxiety, depression, symptom, cross-sectional, survey

## Abstract

**Background:**

The COVID-19 global pandemic has increased the burden of mental illness on Canadian adults. However, the complex combination of demographic, economic, and lifestyle factors and perceived health risks contributing to patterns of anxiety and depression has not been explored.

**Objective:**

The aim of this study is to harness flexible machine learning methods to identify constellations of factors related to symptoms of mental illness and to understand their changes over time during the COVID-19 pandemic.

**Methods:**

Cross-sectional samples of Canadian adults (aged ≥18 years) completed web-based surveys in 6 waves from May to December 2020 (N=6021), and quota sampling strategies were used to match the English-speaking Canadian population in age, gender, and region. The surveys measured anxiety and depression symptoms, sociodemographic characteristics, substance use, and perceived COVID-19 risks and worries. First, principal component analysis was used to condense highly comorbid anxiety and depression symptoms into a single data-driven measure of emotional distress. Second, eXtreme Gradient Boosting (XGBoost), a machine learning algorithm that can model nonlinear and interactive relationships, was used to regress this measure on all included explanatory variables. Variable importance and effects across time were explored using SHapley Additive exPlanations (SHAP).

**Results:**

Principal component analysis of responses to 9 anxiety and depression questions on an ordinal scale revealed a primary latent factor, termed “emotional distress,” that explained 76% of the variation in all 9 measures. Our XGBoost model explained a substantial proportion of variance in emotional distress (*r*^2^=0.39). The 3 most important items predicting elevated emotional distress were increased worries about finances (SHAP=0.17), worries about getting COVID-19 (SHAP=0.17), and younger age (SHAP=0.13). Hopefulness was associated with emotional distress and moderated the impacts of several other factors. Predicted emotional distress exhibited a nonlinear pattern over time, with the highest predicted symptoms in May and November and the lowest in June.

**Conclusions:**

Our results highlight factors that may exacerbate emotional distress during the current pandemic and possible future pandemics, including a role of hopefulness in moderating distressing effects of other factors. The pandemic disproportionately affected emotional distress among younger adults and those economically impacted.

## Introduction

The emergence of the novel coronavirus SARS-CoV-2 in late 2019 and the resulting COVID-19 pandemic have caused social and economic upheaval worldwide. Public health measures to limit the spread of the virus have been linked to negative mental health outcomes, such as depression and anxiety [1-3]. Emotionally distressing symptoms are common, including nonspecific anxiety, fear of illness, loneliness, frustration, and boredom [4], and they are worsened by social isolation due to current lockdown policies [5]. Although these policies effectively limit the spread of infection, a deeper understanding of their effects on mental health is necessary to inform public health interventions.

The COVID-19 pandemic has had disproportionate impacts on some groups compared to others [6]. Millions of North Americans lost work as governments forced closure of businesses and imposed stay-at-home orders. Canadians in the lowest earnings quartile have been particularly affected, accounting for one-half of all job losses in early 2020 [7]. Job insecurity during the pandemic has been associated with symptoms of depression [8]. Furthermore, demographic factors such as female gender [9,10] and younger age [11] have been associated with higher rates of emotional distress during the COVID-19 pandemic. Identifying putative drivers of emotional distress during the COVID-19 pandemic can improve our understanding of population-wise patterns of mental health during a large-scale crisis and aid policy making to support those in need.

Previous literature examining predictive modeling of anxiety and depression has largely focused on classifying patients by anxiety or depression status. Studies predicting anxiety and depression diagnosis from clinical and demographic factors have achieved moderate to high predictive accuracy [12,13], although few studies have predicted symptoms of anxiety and depression at the population level.

The constellation of factors contributing to symptoms of depression and anxiety are appreciably complex. Therefore, meaningful conclusions on the importance of individual factors should be considered in the context of many available data types, as well as over time. We conducted an exploratory study to uncover relationships between predictive factors and self-reported depression and anxiety symptoms in Canadian adults during the COVID-19 pandemic. This study’s first aim was to identify the most important factors predicting a composite score of depression and anxiety symptoms. The second aim was to characterize how associations between demographic and environmental factors and symptom scores changed over time. The third aim was to identify predictors that moderated or exacerbated the effects of others on depression and anxiety by examining two-way variable interactions in our model.

In this study, we applied a flexible decision tree–based machine learning method, eXtreme Gradient Boosting (XGBoost), to model a composite score of depression and anxiety using 50 explanatory factors related to sociodemographic characteristics, substance use, employment, and perceived COVID-19 risk. Data were collected from a cross-sectional survey administered to Canadians between May and December 2020. The XGBoost algorithm allowed inclusion of many input variables and simultaneous consideration of nonlinear and interactive effects between all inputs. We identified the most important predictive factors related to depression and anxiety, and we assessed changes in these effects over time.

## Methods

### Data Collection

Data were collected via repeated cross-sectional surveys between May 8 and December 1, 2020. A total of 6 waves of data were collected using a web-based panel administered by the research and data collection company Delvinia [14]. The sampling waves occurred from May 8 to 12 (n=1005), May 29 to June 1 (n=1002), June 19 to 23 (n=1005), July 10 to 14 (n=1003), September 18 to 22 (n=1003), and November 27 to December 1 (n=1003). Participants were sampled independently in each wave. The overall response rate was 16.1% (6021/38,987).

Quota sampling based on age, gender, and region was used to obtain a sample that is proportional to the English-speaking population of Canada. Canadians aged ≥18 years were eligible. Respondents provided written informed consent electronically prior to participation. Research ethics approval was obtained from the Centre for Addiction and Mental Health research ethics board.

Survey questions included information on demographics, anxiety, depression, substance use, employment changes, perceived risks, and worries related to COVID-19. A full list of variables considered for analysis is included in Table S1 in Multimedia Appendix 1. Respondents’ anxiety levels were captured using the Generalized Anxiety Disorder–7 questionnaire (GAD-7) [15], a validated inventory measuring the frequency of anxiety symptoms over the past 2 weeks. Raw item scores were used, ranging from 0 (not at all) to 3 (nearly every day). Depressive symptoms were measured using 3 modified questions from the Centre for Epidemiologic Studies Depression Scale (CES-D) [16]. Feelings of depression, loneliness, and hopefulness over the past week were reported on a Likert-style scale between 0 (“Rarely or none of the time [less than 1 day]”) and 3 (“Most or all of the time [5-7 days]”). For further details on the included explanatory variables, see Text S1 in Multimedia Appendix 1.

### Data Preparation and Quality Control

Given the strong comorbidity of population-level depression and anxiety symptoms [17], as well as their shared neurobiological underpinnings [18], we first examined pairwise correlations among all anxiety questionnaire items (GAD-7) and the 3 available mood/depression (CES-D) questionnaire items, and we applied principal component analysis (PCA). The questions from both scales were similar in scale. Correlations were high between the GAD-7 and CES-D items, allowing for the combination of items from both questionnaires into a single measure of emotional distress using symptoms of both anxiety and depression.

PCA with varimax rotation was used to reduce the number of mood and anxiety variables needed for modeling while retaining as much information as possible from all anxiety and depression variables. PCA is widely used to identify principal axes of variation in psychometric questionnaires [19,20]; in the absence of a validated method of combining items across the GAD-7 and CES-D, PCA was used to retain the largest amount of useful information in a single score incorporating both scales. To properly account for the ordinal nature of the GAD-7 and CES-D variables, polychoric correlations—measuring associations between ordinal variables assumed to be realizations of underlying latent Gaussian distributions [21]—were used. PCA was applied to our outcomes on all observations prior to train and test spitting.

For our data-inclusive approach, all survey questions were considered for inclusion as model predictors, excluding mood and anxiety variables used in our outcome measure. Questions not asked in all 6 survey waves were excluded. Categorical variables were one-hot encoded (1=yes, 0=no for category membership). “Prefer not to answer” responses were treated as missing (see the *Predictive Modeling* section below).

### Statistical Analysis

#### Predictive Modeling

The XGBoost R package [22,23] was used to train and test gradient-boosted regularized tree-based models. The core XGBoost function predicts outcomes by fitting a series of decision trees, each building upon the information from all previous trees to improve predictive performance. XGBoost was chosen to model anxiety and depression symptoms, as its extremely flexible approach can enable modeling of linear, nonlinear, and interactive effects between all inputs simultaneously, allowing for more insight into complex interdependencies within inputs that may not be captured by simpler regression methods.

We withheld 20% of the observations from model training, randomly selected within each survey wave. Optimal model hyperparameters were selected using a random grid search and 10-fold cross-validation on the remaining 80% of observations.

As our latent outcome of interest was continuous, the root mean squared error was used as a loss function. Out-of-sample predictive performance was tested by computing Pearson correlations between predicted and observed distress values. Squared Pearson correlation coefficients (*r*^2^) were calculated to describe the proportion of variance in the observed outcome captured by the predictive model.

XGBoost imputed missing variables by assigning a default direction to each decision node. In the sensitivity analysis, observations with missing values in any inputs were removed from the model training and validation data sets. Out-of-sample performance was compared between the main model (all observations) versus the sensitivity analysis model (only complete observations).

To assess the improvement in predictive performance of XGBoost over a less complex approach that does not account for interactions and nonlinear effects, least absolute shrinkage and selection operator (LASSO) regression was also tested. Regularized regression methods such as LASSO tend to exhibit improved predictive performance compared to unregularized, traditional regression methods via the introduction of a penalty parameter to control overfitting.

Our LASSO model included the same variables predicting distress and was trained on the same set of observations, excluding observations with missing data. The LASSO regularization penalty parameter was optimized via 10-fold cross-validation. Out-of-sample prediction was compared between the LASSO and XGBoost models trained on complete observations only, as well as on the full model using single imputation with predictive mean matching to impute data for LASSO.

#### Variable Importance and Interactions

To understand the relative contribution of each variable to model predictions, we computed importance of each variable using SHapley Additive exPlanations (SHAP) [24]. SHAP values measure the relative strength of each variable’s marginal contribution to an individual’s predicted outcome value, conditioning on all other explanatory variables for that individual [25].

Overall variable importance was defined as the mean absolute value of all SHAP values for a given variable. Negative SHAP values indicate that predicted distress was reduced by that variable, while positive SHAP values indicate a positive influence on predicted distress. Relationships between predicted values and time were examined via partial dependence plots, adjusted for all other explanatory variables. SHAP values for each two-way interaction between variables were also computed and plotted [26].

In the absence of formal hypothesis tests for interaction SHAP values, and to provide a comparable regression-based framework for the interpretation of our XGBoost-identified interactions, we identified statistically significant two-way variable interactions by fitting separate linear regression models to each pair of input variables. Global *P* values for the overall significance of interaction terms were computed via likelihood-ratio tests. The model with both individual variables plus their interaction was compared to a model with the interaction term removed. Benjamini-Hochberg corrections were applied to all interaction global *P* values to constrain the false discovery rate (FDR) to 5%. Interactions were deemed statistically significant if their FDR-adjusted *P* values were <.05.

All analyses were conducted in R, version 3.6.3 (R Foundation for Statistical Computing; see Text S2 in Multimedia Appendix 1).

## Results

### Survey Respondents

A total of 6021 respondents provided complete surveys for analysis. The characteristics of the respondents are summarized in Table 1. Demographic distributions of age, sex, and region were representative of the English-speaking Canadian adult population [27,28].

**Table 1:** Baseline characteristics of the survey respondents (N=6021).

Characteristic			Responses by survey wave, n (%)													*P* value (Fisher test)
			1 (May 8-12, n=1005)		2 (May 29-June 1, n=1002)		3 (June 19-23, n=1005)		4 (July 10-14, n=1003)		5 (Sept 18-22, n=1003)		6 (Nov 27-Dec 1, n=1003)			
**Region**																>.99
	Alberta	140 (13.9)		140 (14)		140 (13.9)		133 (13.3)		137 (13.7)		141 (14.1)				
	British Columbia	152 (15.1)		146 (14.6)		150 (14.9)		151 (15.1)		148 (14.8)		152 (15.2)				
	Ontario	418 (41.6)		418 (41.7)		415 (41.3)		421 (42)		419 (41.8)		419 (41.8)				
	Quebec/Atlantic Canada	182 (18.1)		190 (19)		192 (19.1)		192 (19.1)		191 (19)		187 (18.6)				
	Saskatchewan/Manitoba	111 (11)		108 (10.8)		104 (10.3)		105 (10.5)		106 (10.6)		102 (10.2)				
	Yukon/Northwest Territories/Nunavut	2 (0.2)		0 (0)		4 (0.4)		1 (0.1)		2 (0.2)		2 (0.2)				
**Age (years)**																>.99
	18-39	394 (39.2)		389 (38.8)		394 (39.2)		388 (38.7)		390 (38.9)		392 (39.1)				
	40-59	306 (30.4)		312 (31.1)		307 (30.5)		309 (30.8)		305 (30.4)		305 (30.4)				
	≥60	305 (30.3)		301 (30)		304 (30.2)		306 (30.5)		308 (30.7)		306 (30.5)				
**Gender**																.62
	Female	498 (49.6)		497 (49.6)		499 (49.7)		492 (49.1)		498 (49.7)		503 (50.1)				
	Male	504 (50.1)		492 (49.1)		501 (49.9)		501 (50)		497 (49.6)		492 (49.1)				
	Other	3 (0.3)		13 (1.3)		5 (0.5)		10 (1)		8 (0.8)		8 (0.8)				
**Has children**																.80
	No	776 (77.2)		766 (76.4)		768 (76.4)		761 (75.9)		769 (76.7)		787 (78.5)				
	Yes	229 (22.8)		236 (23.6)		237 (23.6)		242 (24.1)		234 (23.3)		216 (21.5)				
**Education**																.55
	High school or less	111 (11)		104 (10.4)		129 (12.8)		122 (12.2)		119 (11.9)		99 (9.9)				
	Some post-–high school education	159 (15.8)		165 (16.5)		148 (14.7)		162 (16.2)		147 (14.7)		150 (15)				
	University or college	728 (72.4)		727 (72.6)		720 (71.6)		706 (70.4)		731 (72.9)		742 (74)				
	Prefer not to answer	7 (0.7)		6 (0.6)		8 (0.8)		13 (1.3)		6 (0.6)		12 (1.2)				
**Marital status**																.84
	Married/living with partner	613 (61)		605 (60.4)		622 (61.9)		634 (63.2)		638 (63.6)		653 (65.1)				
	Never married	251 (25)		251 (25)		253 (25.2)		233 (23.2)		239 (23.8)		216 (21.5)				
	Separated/divorced/widowed	128 (12.7)		132 (13.2)		119 (11.8)		122 (12.2)		113 (11.3)		118 (11.8)				
	Prefer not to answer	13 (1.3)		14 (1.4)		11 (1.1)		14 (1.4)		13 (1.3)		16 (1.6)				
**Race/ethnicity**																.88
	White (European, North American)	698 (69.5)		702 (70.1)		691 (68.8)		697 (69.5)		699 (69.7)		691 (68.9)				
	Asian	200 (19.9)		175 (17.5)		201 (20)		188 (18.7)		190 (18.9)		202 (20.1)				
	Black (African, Caribbean, North American)	16 (1.6)		18 (1.8)		19 (1.9)		24 (2.4)		23 (2.3)		13 (1.3)				
	Other	71 (7.1)		78 (7.8)		68 (6.8)		66 (6.6)		60 (6)		70 (7)				
	Not sure/prefer not to answer	20 (2)		29 (2.9)		26 (2.6)		28 (2.8)		31 (3.1)		27 (2.7)				
**Household income (CAD $)^a^**																.61
	Less than 40,000	128 (12.7)		121 (12.1)		136 (13.5)		118 (11.8)		116 (11.6)		110 (11)				
	40,000-79,000	268 (26.7)		236 (23.6)		238 (23.7)		235 (23.4)		247 (24.6)		236 (23.5)				
	80,000-119,000	226 (22.5)		229 (22.9)		220 (21.9)		213 (21.2)		237 (23.6)		241 (24)				
	120,000 or more	217 (21.6)		259 (25.8)		247 (24.6)		252 (25.1)		228 (22.7)		251 (25)				
	Prefer not to answer	166 (16.5)		157 (15.7)		164 (16.3)		185 (18.4)		175 (17.4)		165 (16.5)				
**Locality**																.97
	Rural area	158 (15.7)		164 (16.4)		151 (15)		171 (17)		164 (16.4)		164 (16.4)				
	Suburban area	382 (38)		379 (37.8)		369 (36.7)		365 (36.4)		376 (37.5)		365 (36.4)				
	Urban area	465 (46.3)		459 (45.8)		485 (48.3)		467 (46.6)		463 (46.2)		474 (47.3)				
**Heavy alcohol use (past 7 days)^b^**																.67
	None or light alcohol use	765 (76.1)		753 (75.1)		736 (73.2)		726 (72.4)		744 (74.2)		743 (74.1)				
	Heavy alcohol use	238 (23.7)		247 (24.7)		267 (26.6)		271 (27)		255 (25.4)		257 (25.6)				
	Prefer not to answer	2 (0.2)		2 (0.2)		2 (0.2)		6 (0.6)		4 (0.4)		3 (0.3)				
**Cannabis use**																.18
	No cannabis use	889 (88.5)		869 (86.7)		878 (87.4)		870 (86.7)		881 (87.8)		840 (83.7)				
	Used cannabis	115 (11.4)		130 (13)		124 (12.3)		131 (13.1)		119 (11.9)		160 (16)				
	Prefer not to answer	1 (0.1)		3 (0.3)		3 (0.3)		2 (0.2)		3 (0.3)		3 (0.3)				

^a^CAD $1=US $0.80.

^b^Heavy alcohol use was defined as 5 or more standard drinks for men and 4 or more standard drinks for women in a given day.

### Calculation of Latent Feature Representing Anxiety and Depression

**Figure 1:**
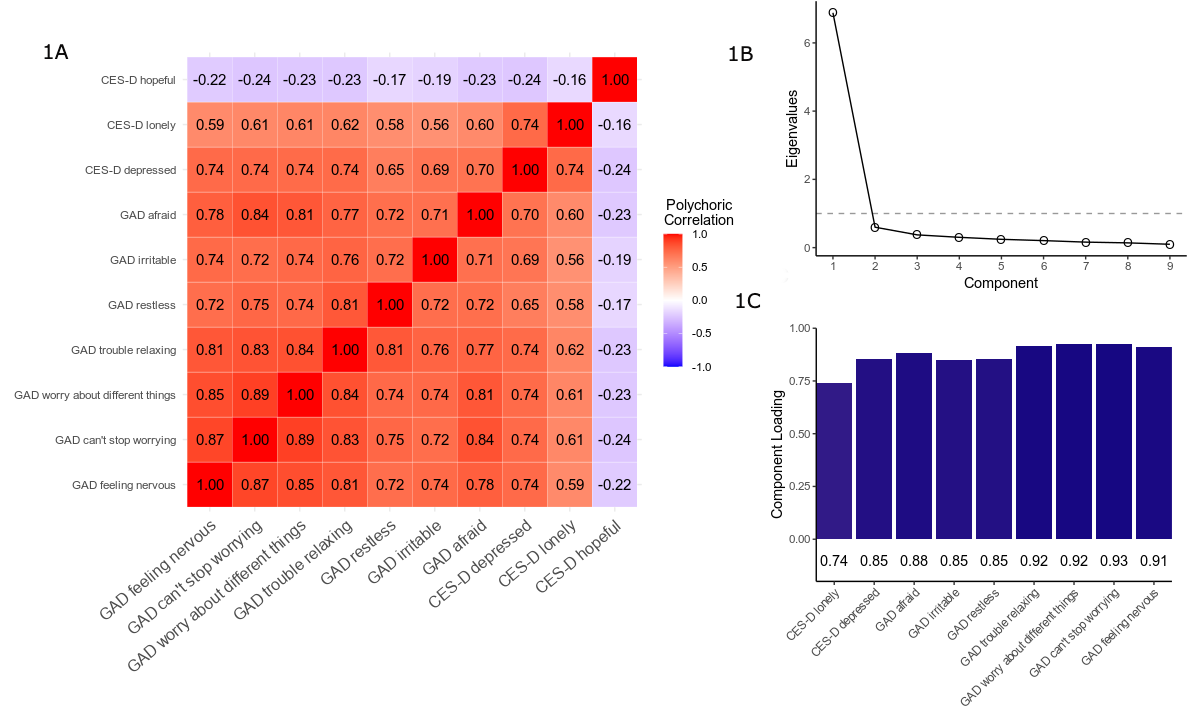
Results of the principal component analysis. (A) Heat map of the polychoric correlations; (B) scree plot of the principal component analysis and loadings onto principal component 1; (C) Variable Loadings onto Principal Component 1. CES-D: Centre for Epidemiologic Studies Depression Scale; GAD: Generalized Anxiety Disorder–7 questionnaire.

The PCA was initially fit using 10 survey items: 7 from the GAD-7 (anxiety) and 3 from the CES-D (depressive symptoms). The polychoric correlations between the anxiety and depression variables are presented in Figure 1A. All items were moderately to strongly positively correlated (*r*=0.55-0.89), with the exception of hopefulness (CES-D item; correlation coefficients –0.18 to –0.27). All items except for hopefulness were loaded strongly onto principal component 1 (PC1) (loadings 0.74-0.93), with hopefulness loading weakly onto PC1 (loading –0.28) and strongly onto principal component 2 (PC2) (loading 0.96); this finding supported our choice to model a single latent outcome combining both scales. Given the weak negative correlation between hopefulness and the remaining mood and anxiety variables, hopefulness was dropped from the PCA and instead included as an explanatory variable in downstream modeling. Distributions of the GAD and CES-D scores and PC1 are included in Figure S1 (Multimedia Appendix 1).

The final PCA included 9 items. In the remainder of this analysis, we refer to PC1 as “emotional distress.” Figure 1B displays a scree plot for this 9-item PCA; the point of inflection occurs at the second PC, indicating that the PCs after the first do not add substantial additional information. The loadings on PC1, emotional distress, are presented in Figure 1C. The item loadings ranged from 0.74 to 0.93. Emotional distress explained 76% of the variance in all 9 variables.

### Fitting of the XGBoost Model and Comparison to LASSO

A total of 50 predictor variables were included in our primary XGBoost model. We included 4819 respondents in the training data set; out-of-sample prediction was tested on the remaining 1202 respondents. For the results of the hyperparameter selection, see Text S3 in Multimedia Appendix 1. The final model explained 38.7% of the variance in distress in the out-of-sample prediction (*r*^2^=0.387). A scatterplot of the predicted and realized values of emotional distress is presented in Figure S2, Multimedia Appendix 1. In training, the *r*^2^ value of the model was 0.394, which is close to the *r*^2^ value for our holdout test set; this indicates that there was no substantial overfitting. For a LASSO model fit to the full test set, *r*^2^ was 0.354 in the withheld validation set, which is slightly lower than the value for XGBoost in predictive accuracy.

**Figure 2:**
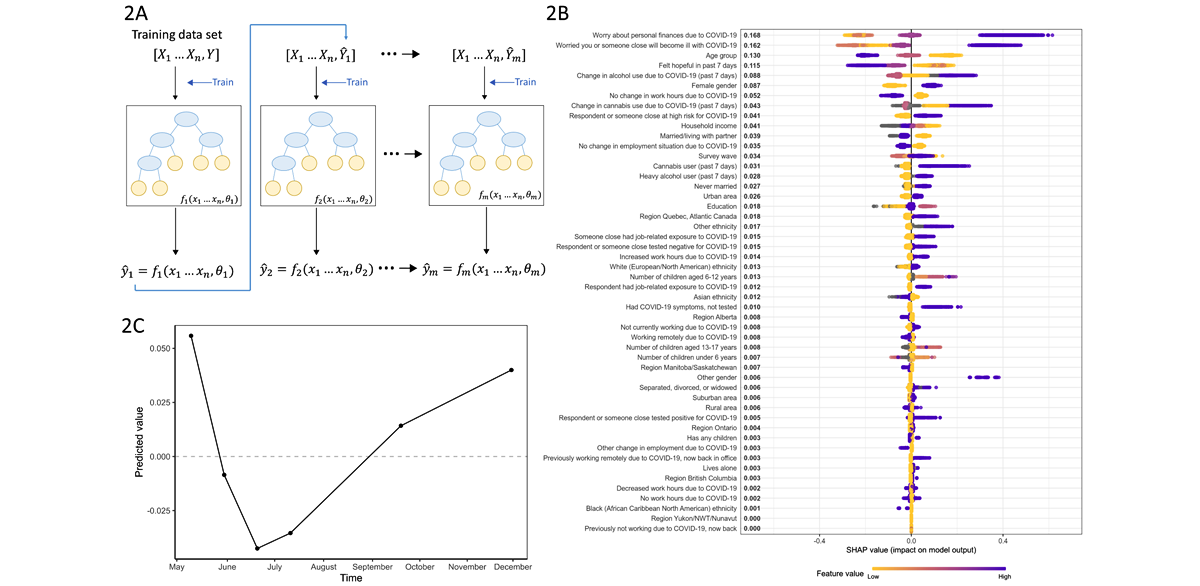
(A) Gradient-boosted tree model training diagram; (B) variable importance plot ranked by mean absolute SHAP value; (C) partial dependence plot of predicted emotional distress values across survey waves. NWT: Northwest Territories; SHAP: SHapley Additive exPlanations.

A graphical depiction of model training using gradient-boosted trees [29] is presented in Figure 2A. A visual representation of the first 6 gradient-boosted trees in the fitted model is included in Figure S3 (Multimedia Appendix 1). A partial dependence plot overlaid with COVID-19 positivity rates [30] is presented in Figure S4 (Multimedia Appendix 1).

To determine if the model fit was sensitive to the XGBoost imputation algorithm for variables with missing values, XGBoost was refit with reoptimized hyperparameters and validated using only observations with complete data (n=3689). On the withheld data, this model explained 36.9% of the variance in emotional distress (*r*^2^=0.369). Results of a LASSO model fit to the same set of complete observations indicated slightly lower performance compared to the XGBoost fit (LASSO *r*^2^=0.346), indicating that distress was reasonably approximated by a linear fit, although at a loss of approximately 2% of the explained variance when nonlinear and interaction relationships were not considered.

### Identification of Variables Most Strongly Associated with Emotional Distress

The SHAP values are presented in Figure 2B. To aid the direct interpretability of our SHAP value analysis, we modeled each questionnaire item as a function of our latent emotional distress outcome using linear regression. Each 1-unit change in our outcome corresponded to a difference in the question response value of between 0.68 and 0.84 (mean 0.78) across all 9 questions, meaning that a SHAP value of 0.61 (the largest value reported for individuals with high levels of reported financial worry) would translate to an average increase of 0.61*0.78=0.48 across the original question scales. Given that each question was measured as integers ranging from 1 to 4—representing an underlying quantitative scale mapping onto the number of recent days when symptoms were experienced—this maximum SHAP value would represent a predicted marginal change of 16% of the entire spectrum of symptom burden across all questions in the holdout test population: 0.48/(4-1)*100.

The variables with the greatest importance, in descending order, were worry about personal finances due to COVID-19 (mean absolute SHAP value=0.168), worry that oneself or loved ones will become ill with COVID-19 (0.162), age group (0.130), hopefulness (0.115), change in alcohol use due to the pandemic (0.088), and female gender (0.087).

For both financial and illness-related worries, low to moderate levels of worry were associated with decreased or average predicted distress, while severe worries increased predicted distress (0.41 for “very worried” about finances; 0.34 for “very worried” about illness). To a lesser extent, greater hopefulness was associated with decreased predicted distress. The relationship between age group and predicted emotional distress was somewhat linear: membership in the youngest age group (18 to 39 years) increased predicted distress, while membership in the age groups of 40 to 59 years and ≥60 years decreased predicted distress. Female gender increased predicted distress, and both increased and decreased alcohol intake compared to prepandemic intake had higher mean SHAP values compared to no change (mean SHAP values of 0.19 for increased use 0.12 for decreased use; –0.07 no change), indicating a nonlinear effect. Change in cannabis use due to the pandemic was directionally similar (mean SHAP values of 0.25 for increased use, 0.11 for decreased use, and –0.02 for no change), although its overall importance was lower.

Following the top 6 ranked variables, we found a heuristic “elbow point” separating the most important variables from those with lesser importance. For all remaining variables, the mean SHAP values were at or below 0.05. Of note, some important variables demonstrated low mean SHAP values, but their range of values was large. Notably, only a small number of respondents answered “other” (nonbinary) for gender identity (n=47); although these responses had a strong influence on their individual predicted values, mean SHAP values for the feature as a whole remained relatively low (mean absolute SHAP value 0.006, range –0.04 to 0.38).

To examine the effects of time, we explored predicted emotional distress values across survey waves. The survey wave variable ranked 14th out of 50 variables with respect to variable importance (mean absolute SHAP value 0.024), indicating that the passing of time was not as influential in our model as other time-independent variables. A partial dependence plot is shown in Figure 2C; when all other variables were held constant, predicted emotional distress was highest in wave 1 (May 8-12; adjusted mean 0.06). After wave 1, the values decreased (wave 2 adjusted mean –0.01). The predicted values decreased most by membership in wave 3 (mean –0.04, June 19-23) and wave 4 (mean –0.03, July 10-14), then increased again in waves 5 and 6 (mean 0.01 for September 18-22 and 0.04 for November 27-December 1).

### Pairwise Interactions of Features in Predicting Emotional Distress

**Figure 3:**
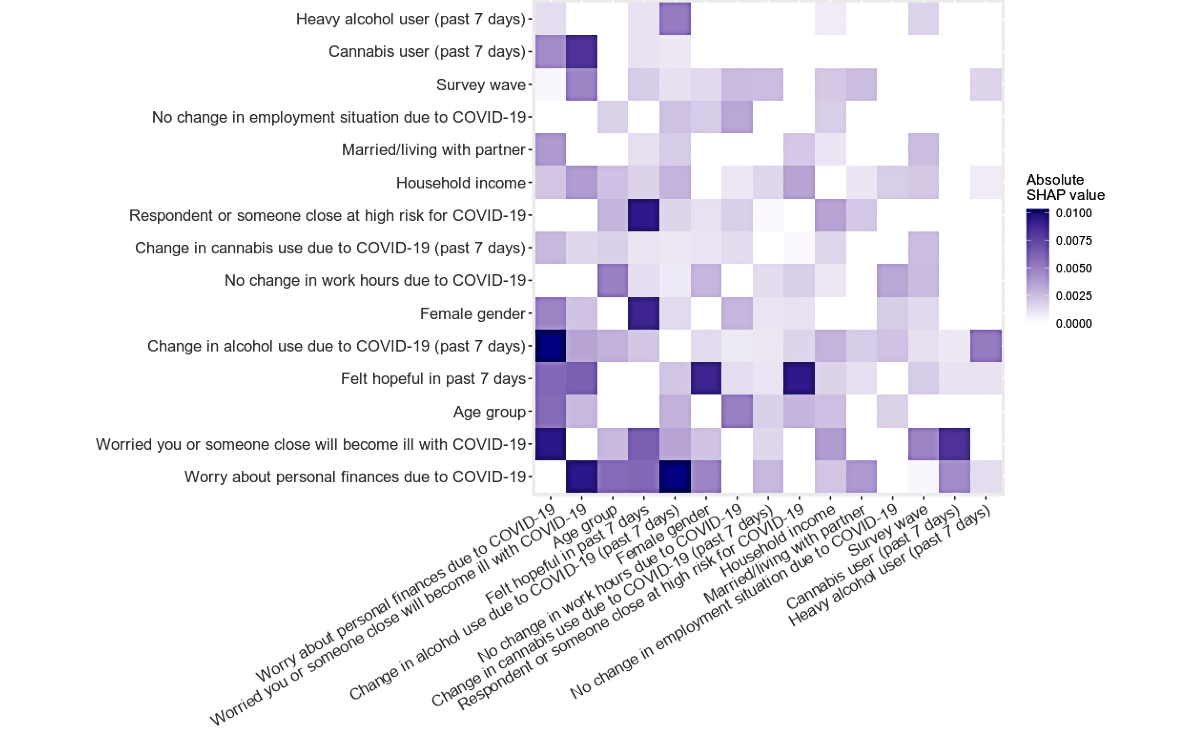
SHAP variable importance of two-way variable interactions for the top 15 most important variables. SHAP: SHapley Additive exPlanations.

We next performed exploratory analysis of the importance of two-way variable interactions in our model. Mean SHAP values for two-way variable interactions from the top 15 most important variables are presented in Figure 3; all interactions are shown in Figure S5 (Multimedia Appendix 1). Overall, interactions between features were not substantially important in determining model predictions; the mean absolute SHAP values for the interaction terms ranged from 0 to 0.01, which represents a maximum of approximately 1/17 of the mean contribution of the most important variable (worry about finances). A total of 5 interactions had SHAP values above 0.008 (the visual elbow point); these top interactions included change in alcohol use × worry about finances (SHAP 0.010), worry about getting COVID-19 × worry about finances (SHAP 0.010), hopefulness × high risk for COVID-19 (0.009), hopefulness × female gender (0.009), and worry about getting COVID-19 × cannabis use (0.008).

**Figure 4:**
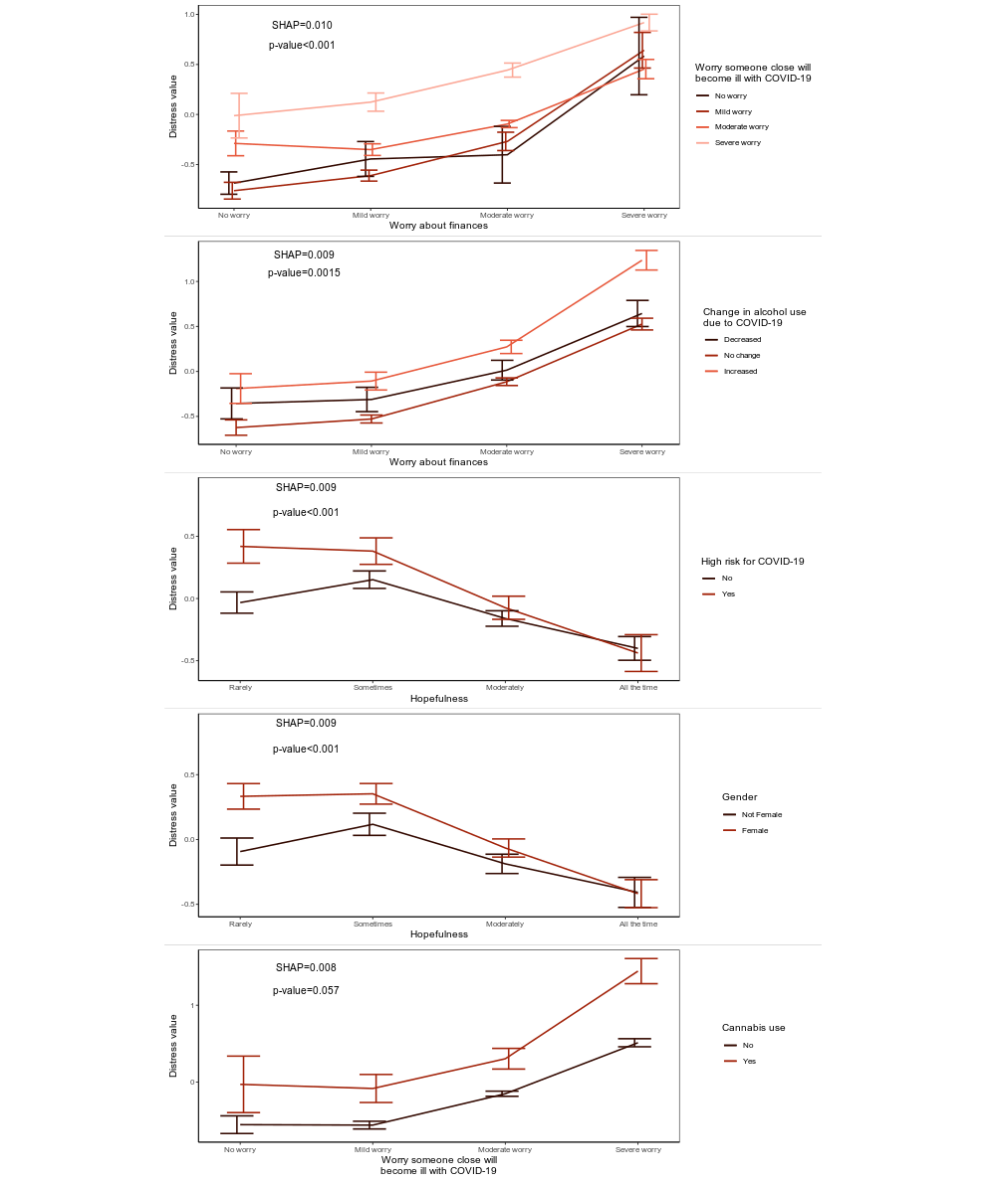
Relationships between pairs of variables in five most important variable interactions and predicted distress.

Figure 4 displays relationships between pairs of variables in the top 5 most important interactions. Plotting both COVID-19–related worries (financial and illness-related) against predicted emotional distress, the distressing effects of illness-related worry were stronger at lower levels of financial worry, where individuals with severe financial worry had the greatest distress regardless of illness-related worries. Additionally, greater hopefulness mitigated differences in distress levels between those who were at high risk for COVID-19 (or had loved ones at high risk) versus those who were not, as well as differences in distress between female and nonfemale respondents. Finally, those who used cannabis had a steeper increase in distress as illness-related worries increased.

To validate our findings, unregularized regression analyses were performed to test the statistical significance of pairwise interaction models with realized distress values. Out of 1225 possible interactions between pairs of explanatory variables in bivariate linear regression on emotional distress, 58 interaction terms had significant associations after Benjamini-Hochberg correction. Of these 58 interactions, 10 involved hopefulness. Of all 5 interactions with SHAP variable importance above the elbow point of 0.008, the top 4 were also statistically significant in the regression analysis (see Table S2 in Multimedia Appendix 1).

## Discussion

### Principal Findings

In this study, we used machine learning to examine factors associated with emotional distress during the COVID-19 pandemic, informed by self-reported levels of anxiety and depression symptoms, using data from a large national survey. We explored relationships between a wide range of sociodemographic characteristics, substance use patterns, and COVID-19–related perceived risks and worries with distress by examining nonlinear patterns in variable importance and by characterizing the importance of variable interactions.

Our findings that the top predictive factors for emotional distress included worries related to COVID-19 are consistent with a recent study of COVID-19–related anxieties [10], suggesting that the participants experienced substantial health and financial concerns. Female gender contributed substantially to increased distress; this finding is consistent with evidence that even before the pandemic, both general anxiety [31] and depression [32] as well as COVID-19–specific anxieties [10] have been shown to be greater in women than in men. Our top predictive features also included change in alcohol use, which showed a nonlinear effect whereby any change (consuming either more or less alcohol compared to before the pandemic) was associated with an increase in emotional distress. This suggests that individual attempts to mitigate above-average levels of pandemic-related distress by drinking less have not been successful and that those who increased alcohol consumption due to the pandemic have experienced subsequent heightened distress.

Hopefulness was only weakly to moderately correlated with other anxiety and depression questionnaire items, and it was loaded onto its own principal component in PCA. These findings suggest that hopefulness may not measure the same latent construct captured by the remaining anxiety and depression survey items. This relative lack of cohesion between hopefulness and the other CES-D questions (feelings of loneliness and depression) in latent variable analysis is supported by previous work finding structural inconsistencies among CES-D items [33,34] Given the sudden and temporary nature of imposed pandemic restrictions, we hypothesized that the hopefulness responses represent a more trait-like positive affect rather than more situationally influenced responses to social isolation, such as lonely and depressed feelings. In predicting emotional distress, hopefulness was the fourth most important contributor, with higher hopefulness decreasing overall predicted distress. Hopefulness has been linked to lower emotional distress [35], and it may indicate greater resilience to adversities experienced during the COVID-19 pandemic.

One main aim was to assess changes in predicted emotional distress over time. The survey wave was not among the strongest contributors to emotional distress, with an importance approximately one seventh of that of the top variable. Despite this, variation in predicted values was present across time; the highest predicted emotional distress was observed during survey wave 1 in May 2020, amid nationwide lockdowns and just after the peak 7-day average case count [30] in Canada’s “first wave” of COVID cases. Predicted values declined through waves 2 to 4, when case counts were decreasing and lockdown policies were relaxed. Waves 5 to 6 saw a second increase in predicted emotional distress levels through the fall and winter of 2020, as Canada began its second wave of cases. This pattern mirrored the trajectory of COVID-19 case counts nationwide [30]. Our findings are consistent with results from the Canadian Community Health Survey, which found that mental health worsened due to the pandemic, increasing from September to December in 2020 [36]. These results suggest that Canadians experienced a spike of emotional distress at the start of the pandemic, amid fearful public health messaging and great uncertainty. Following this, as case counts decreased and lockdown measures were lifted, we speculate that increased optimism or a reduction in the perceived threat of COVID-19 may have lowered collective distress. This summer period was followed by the fall and winter months, when seasonal changes and increasing case counts again led to increased distress.

Finally, we conducted an exploratory analysis to examine the importance of variable interactions in predicting emotional distress. Although the importance of these interactions was relatively low (1/17th that of the most important single variable), they played a role in determining the predicted values. In particular, the distressing effects of severe COVID-19–related worries were most pronounced when other worries were not present. Notably, high hopefulness also mitigated the effect of several other factors that increased predicted distress, including female gender and high risk for COVID-19.

This study has several strengths. First, it was conducted on a large national survey sample that was designed to be representative of the Canadian population in age, gender, and region. Second, few other studies have examined changes in mental health outcomes across time throughout the first 10 months of the COVID-19 pandemic. Third, we employed flexible, interpretable machine learning methods to detect nonlinear and interactive relationships of many predictors of anxiety and depression symptoms.

A limitation of this study was its cross-sectional design, which meant that we were not able to track changes in mental health outcomes within the same individuals longitudinally, nor were we able to determine the temporality or direction of associations. However, the repeated cross-sectional study design allowed us to track patterns in mental health outcomes over time. A second limitation was that the survey was not administered prior to the COVID-19 pandemic, so a direct comparison with prepandemic mental health was not possible. A third limitation was that the survey was administered on the web, meaning that Canadians who are not comfortable with technology may have been less likely to participate; furthermore, the complete response rate was low (16.1%), indicating potential selection bias. However, quota sampling techniques were used to represent the Canadian adult population as accurately as possible in the complete analysis data set, and this response rate is similar to that expected for population surveys of this length administered on the web and without financial incentive [37]. Finally, information on mental health histories or prior clinical diagnoses was not available. However, these findings provide insight into emotional distress of the Canadian population at large, independent of clinical diagnoses.

### Conclusion

Demographic and COVID-19–related factors were associated with a substantial amount of the variation in emotional distress during the global COVID-19 pandemic. These associations were most strongly driven by COVID-19–related fears, namely worries about personal finance and worries about contracting the illness, and were partly mitigated by high levels of hopefulness.

Rates of negative mental health outcomes such as eating disorders [38], substance use [39], overdose [40], and suicide attempts [40] have risen over the course of the COVID-19 pandemic. Although public health policy has been and continues to be vital to curb the spread of SARS-CoV-2, policy makers should also prioritize the provision of population-level supports to address elevated depression and anxiety symptoms among certain groups. Although we cannot infer causation in our study design, our results indicate that initiatives to mitigate financial worry, alleviate illness-related fear, and promote hopefulness may be effective against symptoms of anxiety and depression in the wake of this and potential future pandemics.

### Data Availability

All study data have been made publicly available [41].

## Appendix

Multimedia Appendix 1 Supplementary material.

## Abbreviations

CES-D: Centre for Epidemiologic Studies Depression Scale
FDR: false discovery rate
GAD-7: Generalized Anxiety Disorder–7 questionnaire
LASSO: least absolute shrinkage and selection operator
PCA: principal component analysis
PC1: principal component 1
PC2: principal component 2
SHAP: SHapley Additive exPlanations
XGBoost: eXtreme Gradient Boosting
