# Supplementary material for: Machine Learning–Based Predictive Modeling of Anxiety and Depressive Symptoms During 8 Months of the COVID-19 Global Pandemic: Repeated Cross-sectional Survey Study
Source: JMIR Ment Health. 2021 Nov 17;8(11):e32876. doi: 10.2196/32876 (PMC8601369; doi:10.2196/32876)
Supplement: Multimedia Appendix 1 [file mental_v8i11e32876_app1.docx]

**Multimedia Appendix 1.** Supplementary material.

**Table S1. Summary of data preprocessing: data available in all waves of data collection (waves 1-6)**

| Source Question | Survey Response Options | Variables Used in Predictive Modelling | Coding |
| --- | --- | --- | --- |
| In which province or territory do you currently live? | 1. Alberta 2. British Columbia 3. Manitoba 4. New Brunswick 5. Newfoundland and Labrador 6. Northwest Territories 7. Nova Scotia 8. Nunavut 9. Ontario 10. Prince Edward Island 11. Quebec 12. Saskatchewan 13. Yukon 14. I currently live outside of Canada | Region: Ontario  (option 9) | 1=Yes, 0=No |
|  |  | Region: Alberta  (option 1) | 1=Yes, 0=No |
|  |  | Region: British Columbia  (option 2) | 1=Yes, 0=No |
|  |  | Region: Quebec/Atlantic Canada (option 4, 5, 7, 10, 11) | 1=Yes, 0=No |
|  |  | Region: Saskatchewan/Manitoba  (option 3, 12) | 1=Yes, 0=No |
|  |  | Region: Yukon, Nunavut, Northwest Territories  (option 6, 8, 13) | 1=Yes, 0=No |
| Age group | 1. 18 to 29 years 2. 30 to 39 years 3. 40 to 49 years 4. 50 to 59 years 5. 60 to 69 years 6. 70 years and over 7. Prefer not to answer | Age 18-39  (option 1, 2) | 1=Yes, 0=No |
|  |  | Age 40-59  (option 3, 4) | 1=Yes, 0=No |
|  |  | Age 60+  (option 5, 6) | 1=Yes, 0=No |
| Gender identity | 1. Man 2. Woman 3. Transgender man 4. Transgender woman 5. Two-Spirit 6. Non-binary (genderqueer, gender fluid) 7. Questioning/Not sure of my gender identity 8. Identity not listed 9. Prefer not to answer | Gender=Male  (option 1) | 1=Yes, 0=No |
|  |  | Gender=Female  (option 2) | 1=Yes, 0=No |
|  |  | Gender=Other gender identity  (option 3-8) | 1=Yes, 0=No |
| Including yourself, how many people are currently living in your household? | 1. ___ Enter number 2. Prefer not to answer | Household (option 1) | 1=Lives alone (number in household = 1),  2=Lives with others (number in household > 1) |
| How many children in each of the following categories live in your household? | 1. Under 6 years old: ___ Enter number 2. 6-12 years old: ___ Enter number 3. 13-17 years old: ___ Enter number 4. Prefer not to answer | Number of children under 6  (option 1) | Count |
|  |  | Number of children 6-12  (option 2) | Count |
|  |  | Number of children 13-17  (option 3) | Count |
| What is the highest level of education you have completed? | 1. Did not graduate from high school 2. Completed high school 3. Some post-high school education (college, technical, university, etc.) 4. College diploma / degree 5. University diploma / degree 6. Prefer not to answer | High school or less  (option 1, 2) | 1=Yes, 0=No |
|  |  | Some post-high school education (option 3) | 1=Yes, 0=No |
|  |  | University/College  (option 4) | 1=Yes, 0=No |
| What is your current marital status? | 1. Married 2. Living with a partner 3. Widowed 4. Divorced 5. Separated 6. Never married 7. Prefer not to answer | Married/Living with Partner  (option 1, 2) | 1=Yes, 0=No |
|  |  | Separated/Divorced/Widowed  (option 3, 4, 5) | 1=Yes, 0=No |
|  |  | Never married  (option 6) | 1=Yes, 0=No |
| Which of the following best describes your racial or ethnic group? (Check one only) | 1. Asian – East (e.g., Chinese, Japanese, Korean) 2. Asian – South (e.g., Indian, Pakistani, Sri Lankan) 3. Asian – South East (e.g., Malaysian, Filipino, Vietnamese) 4. Black (Africa, Caribbean, North American) 5. Indigenous (First Nations, Inuit, Métis) 6. Latin American (e.g., Argentinean, Chilean, Salvadoran) 7. Middle Eastern (e.g., Egyptian, Iranian, Lebanese) 8. White (European, North American) 9. Mixed heritage (e.g. Black – North American & White – North American) 10. Other 11. Not sure 12. Prefer not to answer | Ethnicity=East/South East/South Asian  (option 1, 2, 3) | 1=Yes, 0=No |
|  |  | Ethnicity=Black  (option 4) | 1=Yes, 0=No |
|  |  | Ethnicity=White  (option 8) | 1=Yes, 0=No |
|  |  | Ethnicity=Other  (option 5, 7, 9, 10) | 1=Yes, 0=No |
| What is the total household income you and other members of your household received in the year ending December 31st, 2019 before taxes? Please include income FROM ALL SOURCES such as savings, pensions, rent, and unemployment insurance as well as wages. | 1. less than $20,000 2. $20,000 - $39,999 3. $40,000 - $59,999 4. $60,000 - $79,999 5. $80,000 - $99,999 6. $100,000 - $119,999 7. $120,000 - $139,999 8. $140,000 or more 9. Prefer not to answer | Household Income | 1=Less than $40,000  2=$40,000-$79,000  3=$80,000-$119,000  4=$120,000 or more |
| Do you consider yourself to be living in a… | 1. Urban area 2. Suburban area 3. Rural area | Urban Area (option 1) | 1=Yes, 0=No |
|  |  | Suburban Area (option 2) | 1=Yes, 0=No |
|  |  | Rural Area (option 3) | 1=Yes, 0=No |
| Have you, or those close to you (e.g., close relative/friend), tested positive for COVID-19 or are at high risk of COVID-19? (check all that apply) | 1. I, or someone close to me, has tested positive for COVID-19 2. I, or someone close to me, has had symptoms of COVID-19 but has not been tested 3. I, or someone close to me, has been tested for COVID-19 but it was negative (i.e., they did not have COVID-19) 4. I, or someone close to me, is elderly and/or has a health condition that increases the risk of serious illness from COVID-19 5. I have a job that exposes me to high risk of getting COVID-19 6. Someone close to me has a job that exposes them to high risk of getting COVID-19 7. None of the above | I, or someone close to me, has tested positive for COVID (option 1) | 1=Yes, 0=No |
|  |  | Respondent or someone close tested positive for COVID (option 2) | 1=Yes, 0=No |
|  |  | Respondent or someone close tested negative for COVID (option 3) | 1=Yes, 0=No |
|  |  | Respondent or someone close at high risk for COVID (option 4) | 1=Yes, 0=No |
|  |  | Respondent has job-related exposure to COVID (option 5) | 1=Yes, 0=No |
|  |  | Someone close had job-related exposure to COVID (option 6) | 1=Yes, 0=No |
| How worried are you about the impact of COVID on your financial situation? | 1. Very worried 2. Somewhat worried 3. Not very worried 4. Not at all worried | Worry about personal finances due to COVID | 1=Not at all worried  2=not very worried  3=Somewhat worried  4=Very worried |
| How have physical distancing measures due to the COVID-19 pandemic affected your employment situation? (check one only) | 1. I have continued working, but now I am working from home instead of my usual location 2. I am not currently working, or I have been laid off/let go, due to the pandemic 3. I was working from home due to the pandemic, but now I am back working at my usual location outside the home 4. I was previously not working/ laid off/let go due to the pandemic, but now I am back at work with the same or a new employer 5. No change - I have continued working outside my home, as I always did 6. No change - I have continued working from home, as I always did 7. No change -  I was not employed prior to the pandemic (e.g., retired, student, paid leave, recently graduated) and I have remained unemployed 8. Other | Working remotely due to COVID (option 1) | 1=Yes, 0=No |
|  |  | Not currently working due to COVID (option 2) | 1=Yes, 0=No |
|  |  | Previously working remotely due to COVID, now back in office (option 3) | 1=Yes, 0=No |
|  |  | Previously not working due to COVID, now back (option 4) | 1=Yes, 0=No |
|  |  | No change in employment situation due to COVID (option 5, 6, 7) | 1=Yes, 0=No |
|  |  | Other change in employment due to COVID (option 8) | 1=Yes, 0=No |
| On average, how has the number of hours you are working for pay been affected by the COVID-19 pandemic? | 1. Increased a lot 2. Increased somewhat 3. No change 4. Decreased somewhat 5. Decreased a lot | Increased hours  (option 1, 2) | 1=Yes, 0=No |
|  |  | No Change  (option 3, if employed) | 1=Yes, 0=No |
|  |  | Decreased hours  (option 4, 5) | 1=Yes, 0=No |
|  |  | Not currently working  (if responded option 2 or 7 on previous question, then coded as not currently working) | 1=Yes, 0=No |
| How worried are you that you or someone close to you (close relative or friend) will get ill from COVID-19? | 1. Very worried 2. Somewhat worried 3. Not very worried 4. Not at all worried | Worried you or someone close will become ill with COVID | 1=Not at all worried  2=not very worried  3=Somewhat worried  4=Very worried |
| During the PAST 7 DAYS, on how many days did you drink ALCOHOL? | 1. __ Number of days 2. I do not drink alcohol 3. Prefer not to answer | Heavy drinker  (≥1 day in past 7 days drank 4-5+ standard drinks) | 1=Yes, 0=No |
| Past 7 days, how many days did you have 4-5+ std drinks? | 1. __ Number of days 2. Prefer not to answer |  |  |
| In the PAST 7 DAYS, did you drink more ALCOHOL, about the same, or less alcohol overall than you did before the COVID-19 pandemic started? | 1. Drink much more alcohol 2. Drink slightly more alcohol 3. No change 4. Drink slightly less alcohol 5. Drink much less alcohol 6. Prefer not to answer | Change in alcohol use due to COVID | 1=Drank less  2=No change  3=Drank more |
| Past 7 days, how many days did you use cannabis? | 1. __ Number of days 2. I do not use cannabis 3. Prefer not to answer | Cannabis use  (≥1 day in past 7 days used cannabis) | 1=Yes, 0=No |
| In the PAST 7 DAYS, did you use CANNABIS more often, about the same, or less often overall than you did before the COVID-19 pandemic started? | 1. Much more 2. Slightly more 3. No change 4. Much less 5. Slightly less | Change in cannabis use due to COVID | 1=Used less  2=No change  3=Used more |

**Text S1.** Variable Description

For substance use during the pandemic, respondents were asked to indicate the number of days in the past week that they had engaged in the following: any alcohol consumption; heavy episodic alcohol consumption (≥5 standard drinks, or if female, ≥4 standard drinks per day); and any cannabis use. Respondents also reported on a 5-point Likert-style scale whether their alcohol and cannabis use had decreased, stayed the same, or increased during the COVID-19 pandemic.

To aid interpretability, substance use variables were re-coded as follows: respondents were classified as engaging in heavy alcohol use if they reported consuming more than 5 or more drinks for men/other genders, or 4 or more drinks for women on a single occasion in the past week. Respondents were classified as engaging in cannabis use if they reported any cannabis use in the past week.

Perceived risks related to the pandemic were reported on two items, “How worried are you about the impact of COVID-19 on your personal financial situation?” and “How worried are you that you or someone close to you (close relative or friend) will get ill from COVID-19?”. Responses were reported on a four-point scale from “Not at all worried” to “very worried”. Respondents also reported whether they or loved ones were at high risk, had exhibited symptoms or tested positive for COVID-19, and whether they had experienced changes to their employment situation due to social distancing measures as binary yes/no responses.

**Text S2.** R packages

XGBoost and LASSO models were trained and validated using the xgboost and glmnet packages, respectively. SHAP values were calculated using the SHAPforxgboost package.

**Text S3.** Hyperparameter Selection

For our main model including all observations, the following hyperparameters were selected from randomized grid search: eta (learning rate) = 0.12, max_tree_depth (maximum tree depth) = 2, min_child_weight (minimum child node size) = 8, subsample (subsample ratio of training instances) = 0.5, colsample_bytree (subsample ratio of columns when constructing a tree) = 0.9, gamma (minimum loss reduction required to further split on a leaf node) = 1, lambda (L2 regularization parameter) = 0, alpha (L1 regularization parameter) = 2, and nrounds (number of rounds) =200.

For our second model on only complete observations, hyperparameters selected from grid search were eta = 0.22, max_depth = 2, min_child_weight = 6, subsample = 0.7, colsample_bytree = 0.8, gamma = 1, lambda = 0, alpha = 3, and nrounds =100.

**Figure S1. Distributions and scatter plots of GAD-7 and CES-D scores, and PC1**

**
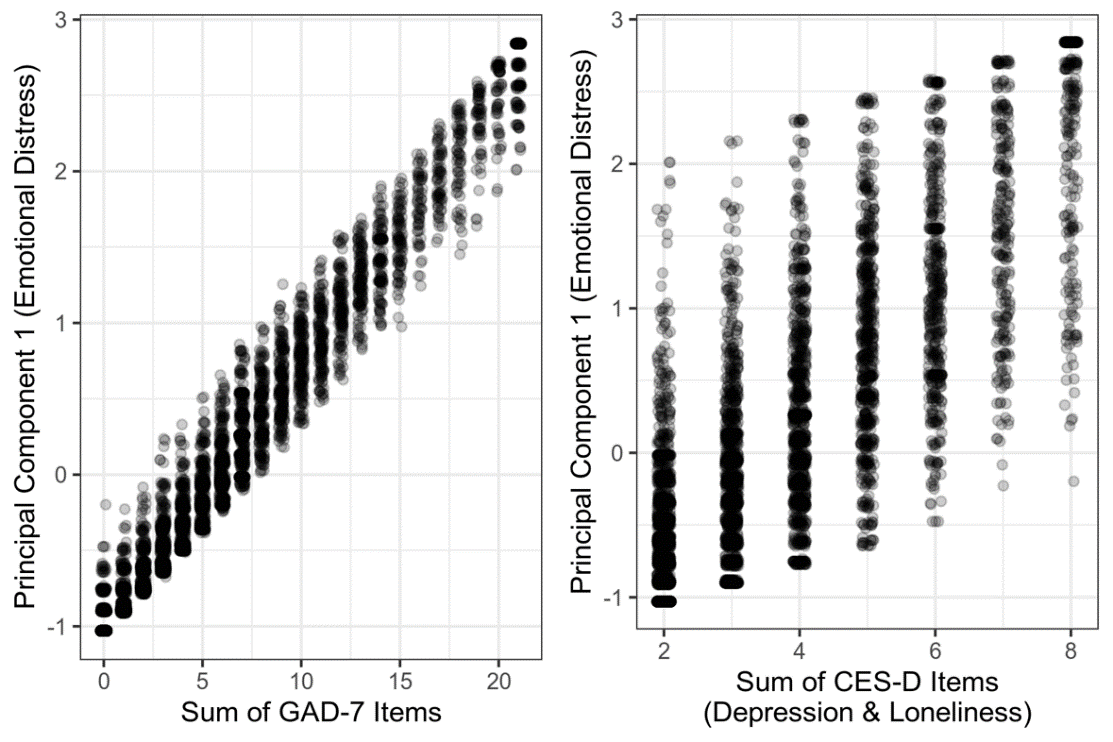
**

A

**
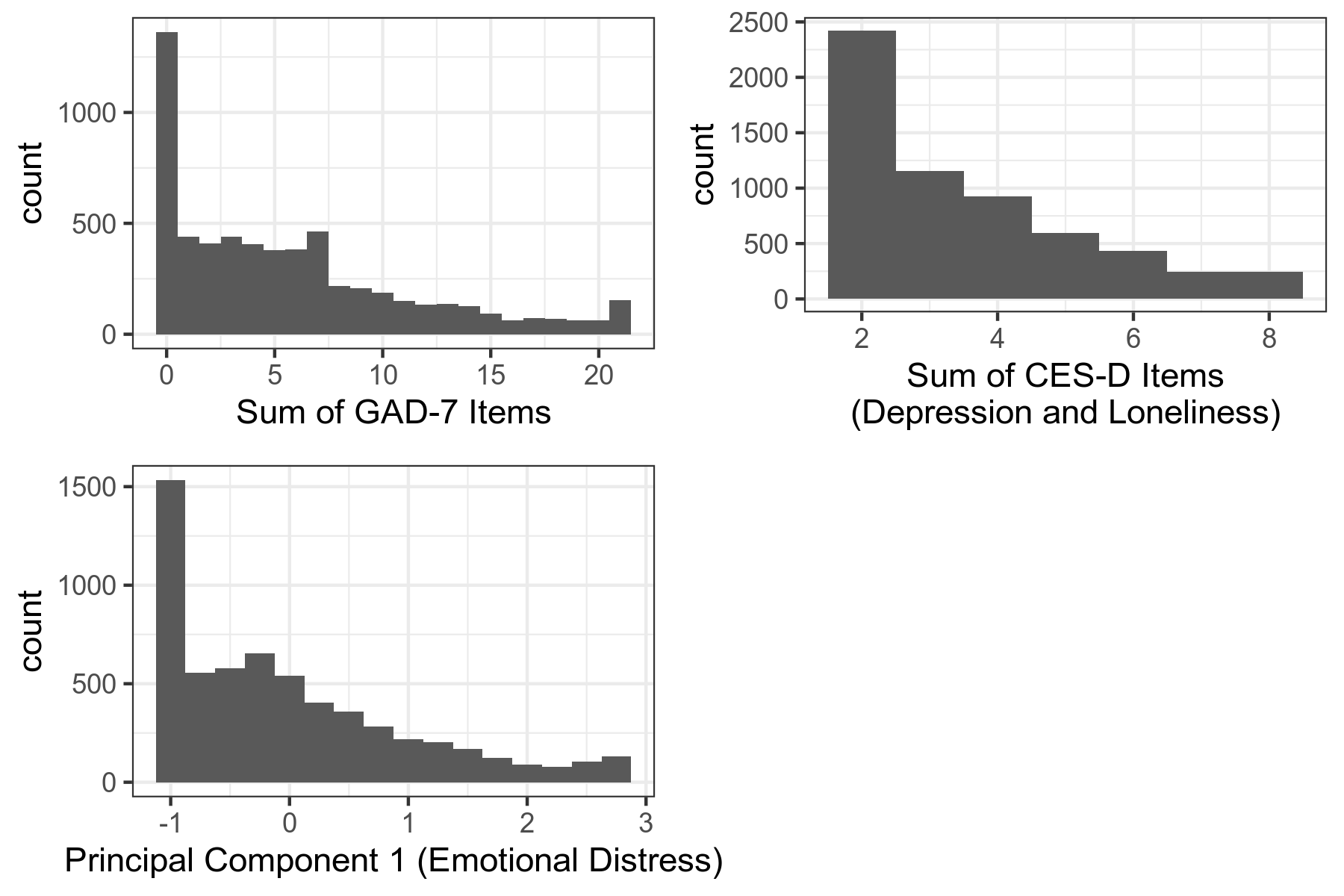
**

B

Figure A is a scatter plot of principal component values against GAD-7 and CES-D scores. Vertical jitter and transparent points have been added to better show distributions of points in each category.

Figure B depicts the distribution of GAD-7, CES-D, and principal component 1 scores in the full dataset.

**Figure S2. Scatter Plot of Realized vs Predicted Emotional Distress**


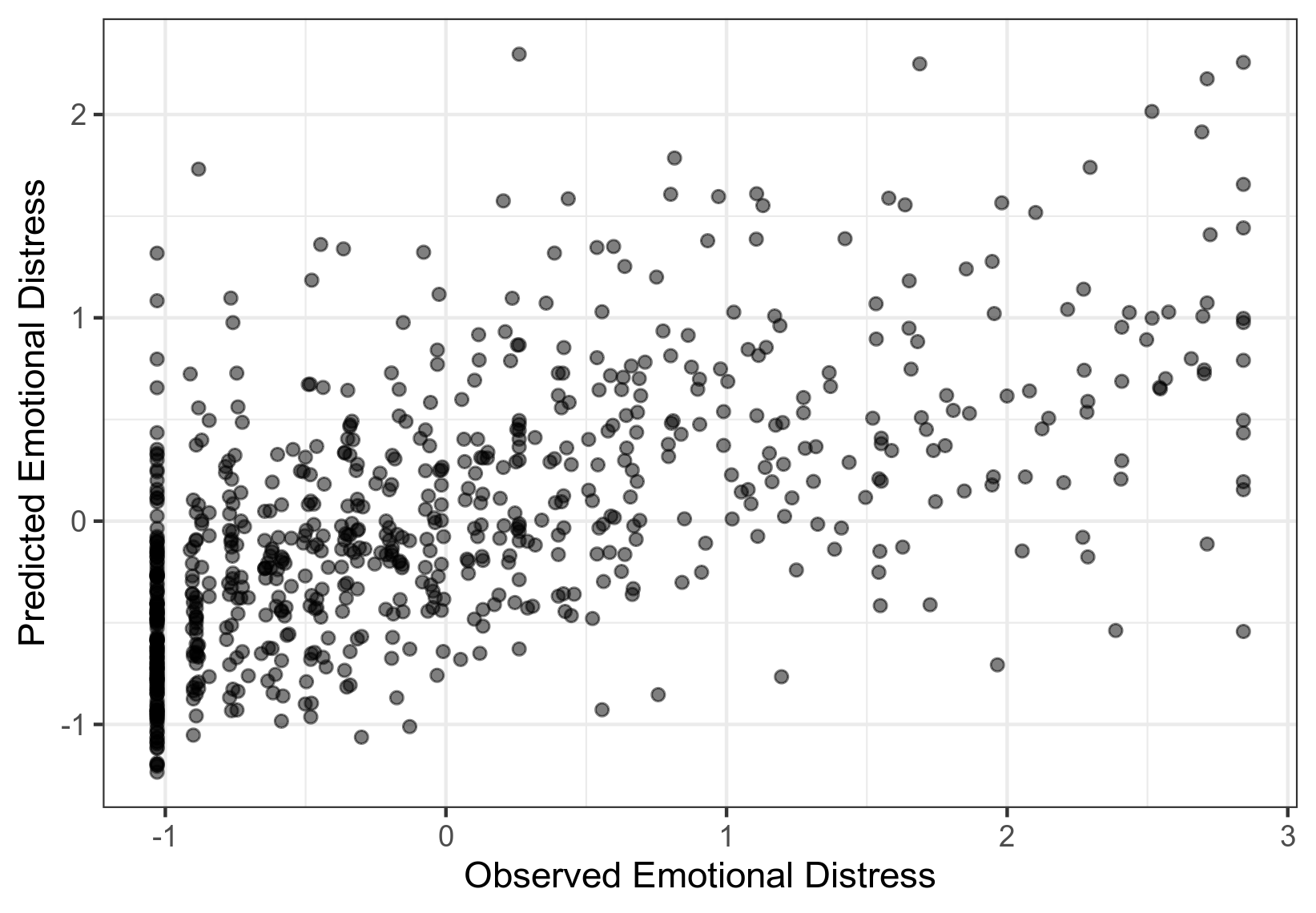


**Figure S3. Gradient-Boosted Tree Model Diagram; First Six Trees in Fitted XGBoost Model**

**
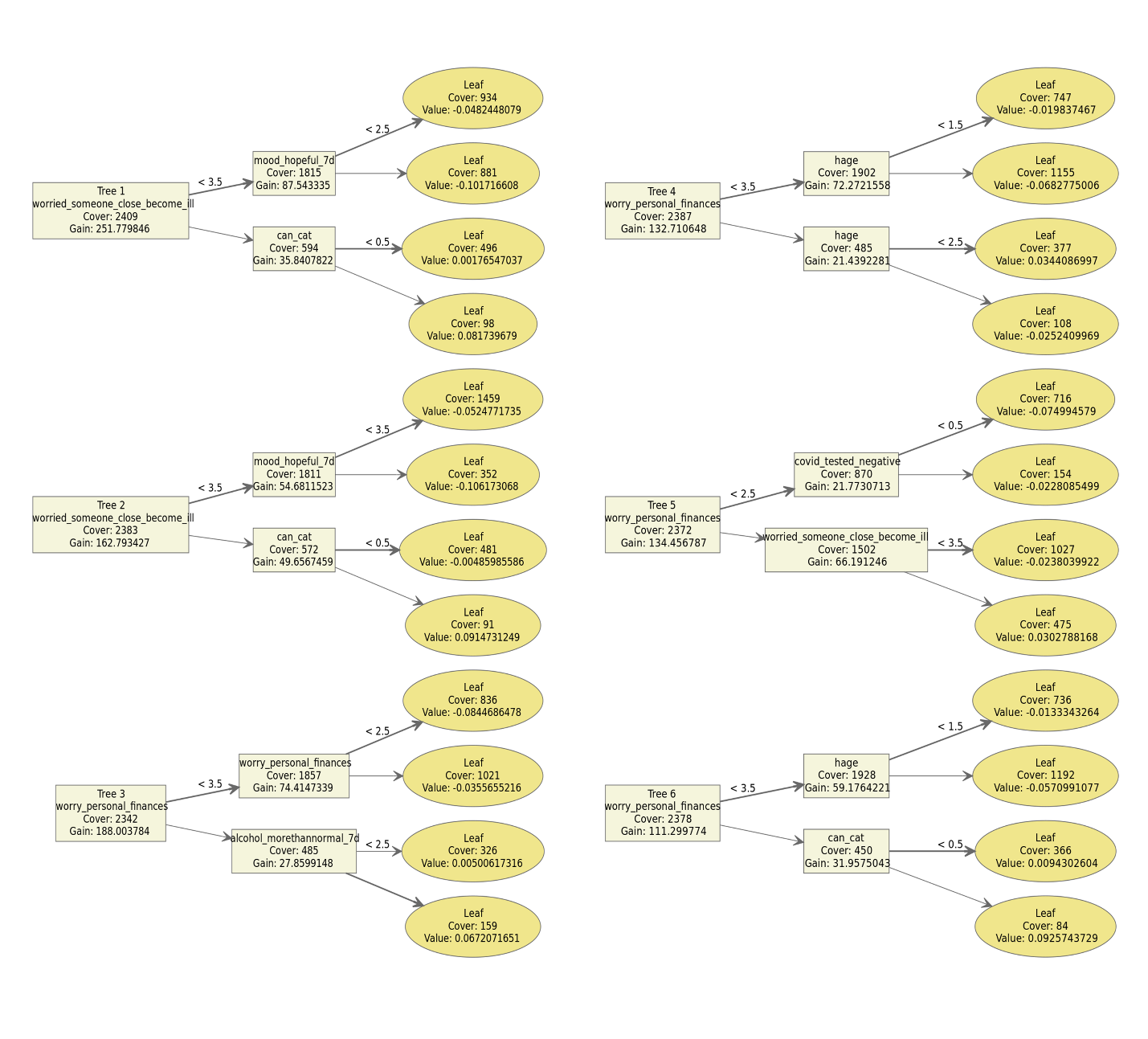
**

**Figure S4. Plot of Predicted Values against COVID case counts**

**
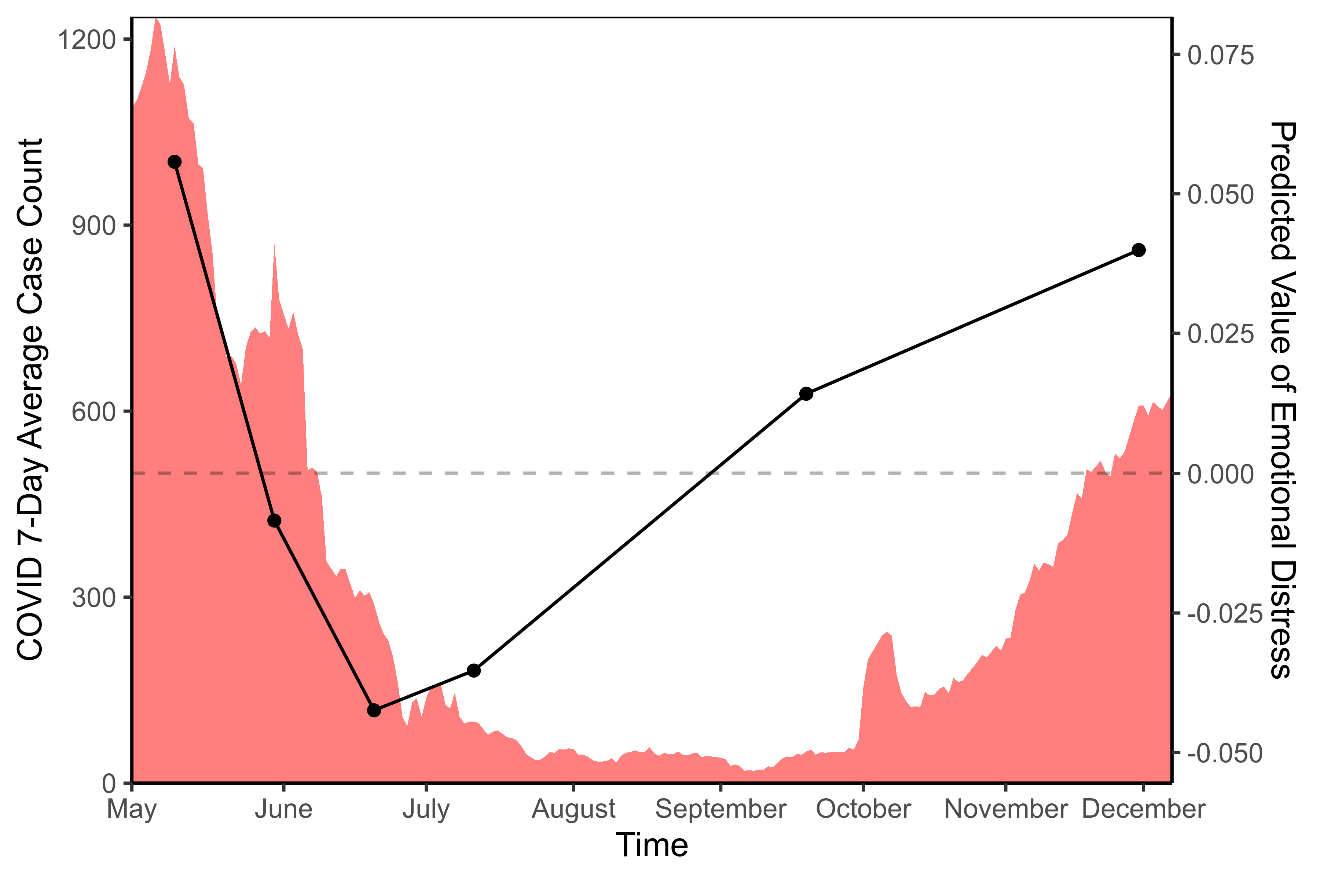
**

**Figure S5. SHAP variable importance of all two-way variable interactions**


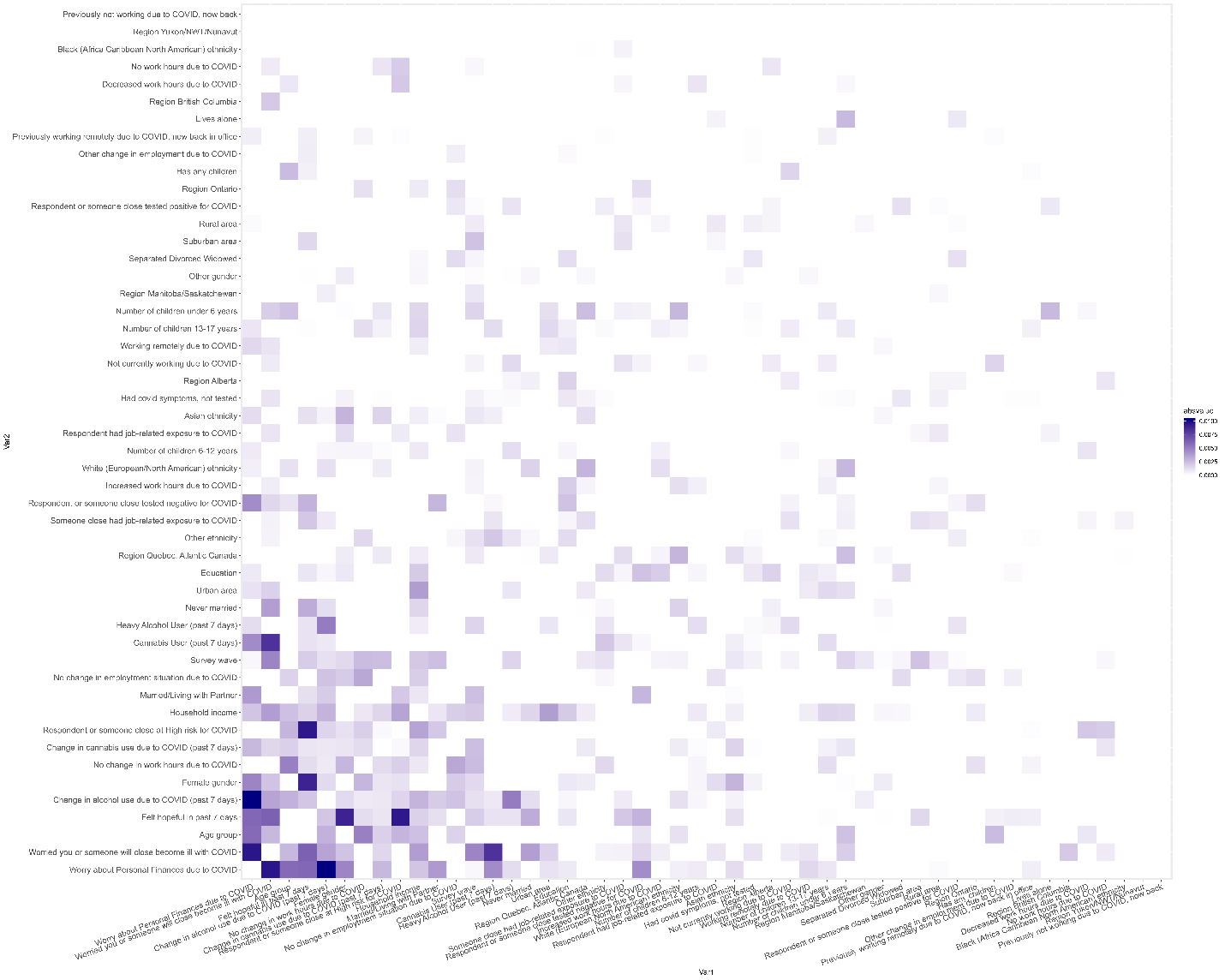


**Table S2. Bivariable linear regression models with interaction terms, on categorical model variables**

| Interaction Model* | *p*-value** | |
| --- | --- | --- |
| Distress ~ No change in employment situation due to COVID * Not currently working due to COVID | 1.765×10^-11^ | |
| Distress ~ Respondent or someone close at High risk for COVID * Felt hopeful in past 7 days | 2.421*10^-11^ | |
| Distress ~ No change in employment situation due to COVID * No change in work hours due to COVID | 1.412*10^-10^ | |
| Distress ~ Lives alone * Separated Divorced Widowed | 2.031*10^-10^ | |
| Distress ~ Worried you or someone will close become ill with COVID * Worry about Personal Finances due to COVID | 1.136*10^-09^ | |
| Distress ~ Has any children * Not currently working due to COVID | 1.018*10^-07^ | |
| Distress ~ Age group * Number of children under 6 years | 2.415*10^-07^ | |
| Distress ~ Felt hopeful in past 7 days * Asian ethnicity | 2.417*10^-07^ | |
| Distress ~ Felt hopeful in past 7 days * White (European/North American) ethnicity | 2.504*10^-07^ | |
| Distress ~ Change in alcohol use due to COVID (past 7 days) * Change in cannabis use due to COVID (past 7 days) | 8.311*10^-07^ | |
| Distress ~ Cannabis Use (past 7 days) * Change in cannabis use due to COVID (past 7 days) | 8.311*10^-07^ | |
| Distress ~ Has any children * No change in work hours due to COVID | 1.608*10^-06^ | |
| Distress ~ Female gender * Felt hopeful in past 7 days | 6.300*10^-06^ | |
| Distress ~ Age group * Not currently working due to COVID | 6.658*10^-06^ | |
| Distress ~ No change in work hours due to COVID * White (European/North American) ethnicity | 6.658*10^-06^ | |
| Distress ~ Age group * No change in work hours due to COVID | 1.350*10^-05^ | |
| Distress ~ Change in alcohol use due to COVID (past 7 days) * Heavy Alcohol Use (past 7 days) | 1.582*10^-05^ | |
| Distress ~ Heavy Alcohol Use (past 7 days) * Felt hopeful in past 7 days | 1.582*10^-05^ | |
| Distress ~ No change in work hours due to COVID * Worry about Personal Finances due to COVID | 3.881*10^-05^ | |
| Distress ~ Cannabis Use (past 7 days) * Worry about Personal Finances due to COVID | 0.00024 | |
| Distress ~ No change in work hours due to COVID * Asian ethnicity | 0.00097 | |
| Distress ~ Someone close had job-related exposure to COVID * Felt hopeful in past 7 days | 0.001 | |
| Distress ~ Respondent had job-related exposure to COVID * Worried you or someone will close become ill with COVID | 0.001 | |
| Distress ~ Working remotely due to COVID * Has any children | 0.0012 | |
| Distress ~ Not currently working due to COVID * Number of children 13-17 years | 0.0015 | |
| Distress ~ Not currently working due to COVID * Never married | 0.0015 | |
| Distress ~ Change in alcohol use due to COVID (past 7 days) * Worry about Personal Finances due to COVID | 0.0015 | |
| Distress ~ No change in work hours due to COVID * Number of children under 6 years | 0.002 | |
| Distress ~ Someone close had job-related exposure to COVID * Worried you or someone will close become ill with COVID | 0.002 | |
| Distress ~ Not currently working due to COVID * Respondent had job-related exposure to COVID | 0.0047 | |
| Distress ~ Household income * Worry about Personal Finances due to COVID | 0.0047 | |
| Distress ~ Respondent or someone close tested negative for COVID * Felt hopeful in past 7 days | 0.005 | |
| Distress ~ Change in cannabis use due to COVID (past 7 days) * Worry about Personal Finances due to COVID | 0.0055 | |
| Distress ~ Working remotely due to COVID * Number of children 13-17 years | 0.0057 | |
| Distress ~ Has any children * Separated Divorced Widowed | 0.0059 | |
| Distress ~ No change in work hours due to COVID * Number of children 13-17 years | 0.0071 | |
| Distress ~ Number of children 13-17 years * Felt hopeful in past 7 days | 0.0072 | |
| Distress ~ Lives alone * Married/Living with Partner | 0.0084 | |
| Distress ~ Heavy Alcohol Use (past 7 days) * No change in work hours due to COVID | 0.011 | |
| Distress ~ Respondent or someone close tested positive for COVID * Lives alone | 0.012 | |
| Distress ~ Female gender * White (European/North American) ethnicity | 0.012 | |
| Distress ~ Respondent or someone close at High risk for COVID * No work hours due to COVID | 0.013 | |
| Distress ~ Never married * White (European/North American) ethnicity | 0.015 | |
| Distress ~ Not currently working due to COVID * White (European/North American) ethnicity | 0.016 | |
| Distress ~ Respondent or someone close at High risk for COVID * Worried you or someone will close become ill with COVID | 0.019 | |
| Distress ~ Age group * Has any children | 0.023 | |
| Distress ~ Respondent or someone close tested negative for COVID * Number of children under 6 years | 0.023 | |
| Distress ~ Female gender * Asian ethnicity | 0.036 | |
| Distress ~ Respondent or someone close tested negative for COVID * Heavy Alcohol Use (past 7 days) | 0.036 | |
| Distress ~ Change in alcohol use due to COVID (past 7 days) * Previously not working due to COVID, now back | 0.039 | |
| Distress ~ Other gender * Felt hopeful in past 7 days | 0.04 | |
| Distress ~ Respondent or someone close tested positive for COVID * Heavy Alcohol Use (past 7 days) | 0.043 | |
| Distress ~ Household income * Number of children 13-17 years | 0.043 | |
| Distress ~ Never married * Asian ethnicity | 0.043 | |
| Distress ~ Had covid symptoms, not tested * No change in work hours due to COVID | 0.046 | |
| Distress ~ Education * Felt hopeful in past 7 days | 0.047 | |
| Distress ~ Someone close had job-related exposure to COVID * Black (Africa Caribbean North American) ethnicity | 0.05 | |
| Distress ~ Household income * Separated Divorced Widowed | 0.05 | |
| *All interaction models included both single variables separately, and their interaction term. Global *p*-values for the interaction term are reported from likelihood-ratio tests.  ***p*-values adjusted via the Benjamini-Hochberg correction for controlling false discovery rate. | |  |
